# ECG changes and their utility in adult Vietnamese patients with non-severe dengue

**DOI:** 10.1186/s12879-026-12926-2

**Published:** 2026-02-26

**Authors:** Hoai Thi Thu Nguyen, Juliette Besson, Ana Bonell, Annette Fox, Natenapa Chimjinda, Mavuto Mukaka, Kinh V. Nguyen, Walter R. Taylor

**Affiliations:** 1 Vietnam National Heart Institute, Bach Mai hospital, Hanoi, Vietnam; 2 VNU - University of Medicine and Pharmacy, Hanoi, Vietnam; 3 Center for Primary Care and Public Health, University of Lausanne, Lausanne, Switzerland; 4 Oxford University Clinical Research Unit, Hanoi, Vietnam; 5 Medical Research Unit The Gambia at London School of Hygiene and Tropical Medicine, Banjul, Gambia; 6 Mahidol Oxford Clinical Tropical Medicine Research Unit, Bangkok, Thailand; 7 Centre for Tropical Medicine and Global Health, University of Oxford, Oxford, UK; 8 National Hospital for Tropical Diseases, Hanoi, Vietnam

**Keywords:** Dengue, Electrocardiogram, Arrythmia, Bradycardia, Myocarditis, Vietnam

## Abstract

**Background:**

Dengue may affect the heart, manifesting as asymptomatic electrocardiographic (ECG) abnormalities to fulminant myocarditis. The role of routine ECG monitoring in dengue management remains unclear. We, therefore, studied ECG changes in patients with non-severe dengue.

**Methods:**

In this observational study, we performed and interpreted 12-lead ECGs (admission, discharge and follow up) on hospitalised Vietnamese adults with confirmed dengue and measured the PR, QRS, and Fridericia-corrected QT intervals (QTcF).

**Results:**

A total of 330 ECGs were performed in 136 patients (64 females; median age, 24 years; range, 16–72 years). Of these, 132 patients had WHO-defined non-severe dengue and 4 had severe dengue. Elevated CK-MB fractions (> 25 IU/L) were detected in 64 of 134 patients (47.8%). The most common ECG abnormality was sinus bradycardia (< 60 beats/min), with 54 episodes occurring in 39 patients (28.7%), most frequently on illness day 10, 9/34 (26.5%) patients. The mean QTcF increased during the first 10 illness days in parallel with a decrease in mean heart rate; only 3 females (461–501 ms) and 1 male (460 ms) had sex-specific QTcF prolongation (≥ 460 ms female, ≥ 450 ms male). Female sex and illness day were significant predictors of QTcF variability over time. Pathological T-wave inversion in the inferior (II, III, aVF) and lateral chest (V5, V6) leads were observed in 12 patients (8.8%), and were mostly transient.

**Conclusions:**

ECG changes associated with nonsevere dengue in predominantly young Vietnamese adults without comorbidities were mostly mild, non-specific, and transient with limited impact on the QTcF interval. Routine ECGs in non-severe dengue in our setting did not provide clinically actionable information.

**Supplementary Information:**

The online version contains supplementary material available at 10.1186/s12879-026-12926-2.

## Background

Dengue is a mosquito-borne viral infection caused by four serotypes of the Flavivirus genus, transmitted primarily by Aedes aegypti. Most infections are self-limiting non-specific or exanthematous febrile illnesses but ~ 5% progress to severe dengue during the critical phase, typically days 3–7 of illness or within 48 h after fever resolution. Severe dengue, as defined by the WHO (2009), involves plasma leakage, shock, severe hemorrhage, or organ dysfunction such as myocarditis or hepatitis, with reported fatality rates up to 5.6% [1–3].

Cardiac manifestations have been reported with varying frequencies in heterogenous dengue patients, from < 10% rising up to 60% in hospitalised patients [2, 4–10]. The pathophysiology of dengue-induced cardiac dysfunction involves multiple factors including endothelial activation and vascular leakage, reduced preload/hypotension, changes in calcium homeostasis, electrolyte imbalance, bleeding in the conduction system, and viral and/or immune mediated myocarditis [11]. Dengue-induced myocarditis is well described and supported by increases in creatine kinase-MB (CKmb) fraction (in ~ 33%) and cardiac troponins (in ~ 25–30%) [8, 10, 12, 13], myocardial inflammation on cardiac magnetic resonance imaging (MRI) [14], interstitial oedema of the myocardium and myocardial necrosis on histology [15], and dengue virus detection by immunohistochemistry in heart muscle [16].

ECG abnormalities in both non-severe and severe dengue are broad, nonspecific and generally resolve over time [2, 7, 9]. Several series have reported mostly normal ECGs, regardless of disease severity or the presence of myocarditis and one Brazilian study reported a mean increase of 12 ms in the Bazett’s corrected QT interval vs. normal controls [12, 17]. However, some recent studies suggest an association between abnormal ECG changes and disease severity, with a trend toward more frequent ECG abnormalities as dengue severity increases [2, 9, 13, 18]. The most common dengue-associated arrhythmias include sinus bradycardia and atrioventricular block (AVB), while other miscellaneous abnormalities such as atrial or ventricular ectopic beats and atrial fibrillation appear less frequently [2, 4, 6, 8, 9, 12, 19–23]. Signs of myocardial injury, such as ST segment elevation and T wave inversion, are typically observed in patients with severe dengue — without corresponding echocardiographic features [27] — and are rare in non-severe cases [9]. Thus, the role of routine ECGs in detecting dengue-related cardiac complications in patients with non-severe dengue does not appear essential, particularly in resource-limited settings. Nevertheless, no study has reported data on ECG abnormalities in this population in Vietnam. We, therefore, aimed to characterise the nature and frequency of ECG abnormalities in adult Vietnamese patients with non-severe dengue and explore their potential clinical significance.

## Methods

### Study site & participants

The study took place from September to November 2008 at the National Hospital for Tropical Diseases (NHTD) and enrolled 143 dengue-confirmed patients, based on any one of the following tests: positive dengue polymerase chain reaction (PCR)/or dengue non-structural protein 1 (NS1) antigen, paired serology showing an immunoglobulin M (IgM) seroconversion, a rising IgM titre of ≥ 20%, an immunoglobulin G (IgG) seroconversion, and a 4-fold rise in IgG titres [24].

### Study procedures

**Fig. 1:**
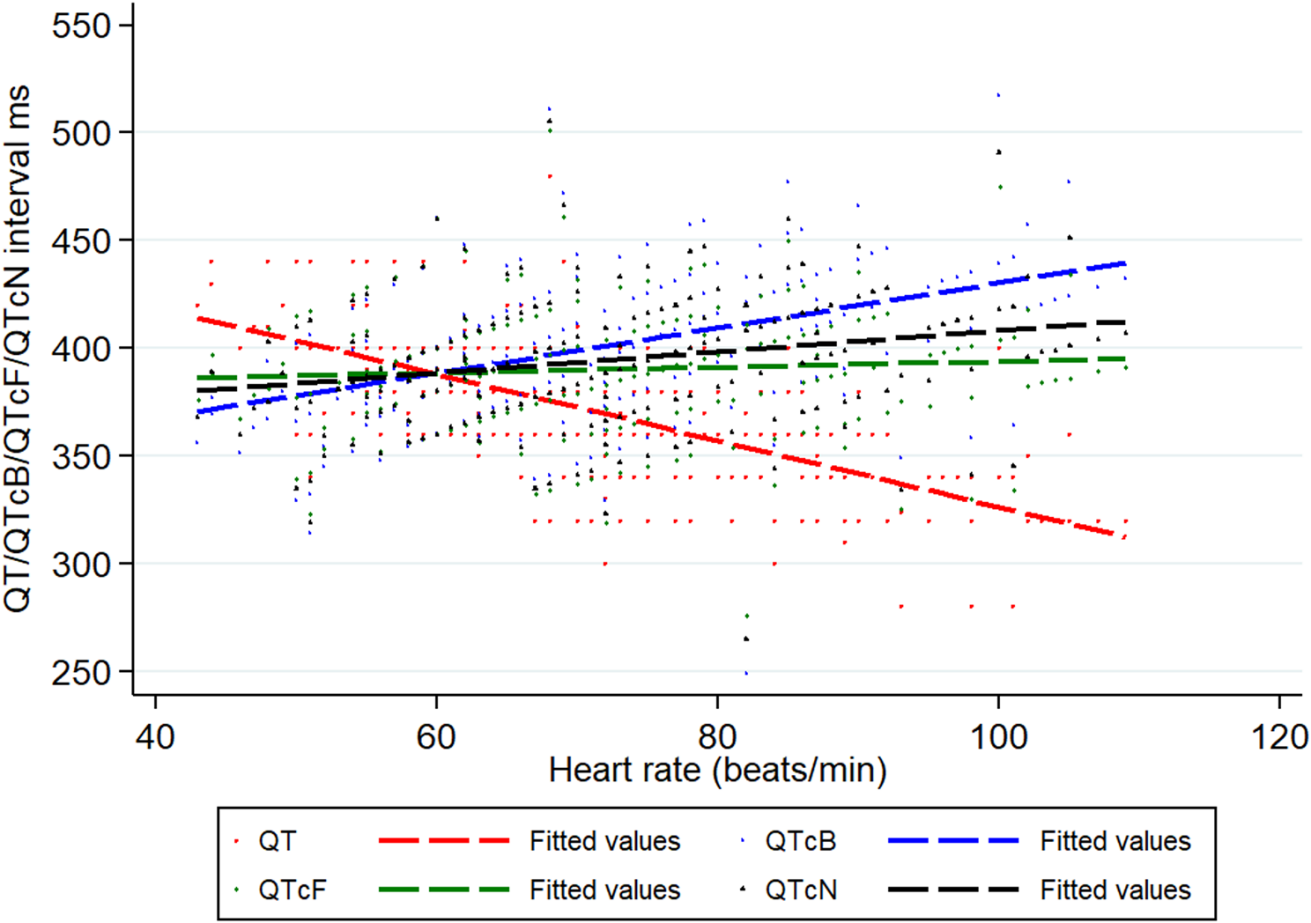
The relationships between the QT and corrected QT intervals and the heart rate. The adjusted R^2^ for the different QT intervals and heart rate were: (i) QT 0.4330 (*p* < 0.001), (ii) QTcB 0.2067 (*p* < 0.001), (iii) QTcN 0.0557 (*p* < 0.001), and (iv) QTcF 0.0018 (*p* = 0.209), confirming the QTcF had the least association

12-lead ECGs (Nihon Kohden machines, Tokyo, Japan) were performed on the day of admission, at discharge, and follow up (generally 2 to 4 weeks later). The paper speed was 25 mm/s and the height calibrated at 10 mm/mV. ECGs were scanned (pdf) and read by the team cardiologist (HTTN) and two experienced physicians (AB, WRT), without the use of on-screen callipers for interval measurements. The findings were recorded onto a standard case report form: rate, rhythm, bundle branch block (BBB), PR, QRS, QT and the Fridericia corrected QT intervals, P, T and ST wave morphology, and left and right ventricular hypertrophy. The Fridericia QT interval, QT/(RR)^0.3^, was chosen because this gave the least correlation (adjusted R^2^ = 0.0018, *p* = 0.209) between the uncorrected QT interval and heart rate compared to the Bazett formula, QT/(RR)^0.5^, and a QT correction formula proposed for malaria QT/(RR)^0.4^ [25] (Fig. 1).

### Definitions

We used widely accepted normal ranges for the (a) ECG intervals: (i) PR 120–200 ms, (ii) QRS 70–110 ms, (b) definitions of sinus bradycardia (< 60/min) and sinus tachycardia (> 100/min), and (c) ECG voltage criteria of left ventricular hypertrophy: S wave V1/V2 + R wave in V5/V6 > 35 mm. For the definition of QTc prolongation, we chose the pragmatic criteria suggested by the American Heart Association of ≥ 450 ms for males and ≥ 460 ms for females [26].

T wave inversion was considered pathological if it occurred in any two of the leads I, II, III, aVF, V5 or V6; an isolated T wave inversion in lead III was not considered pathological as it may be inverted or biphasic as a normal variant. Normal limits of ST elevation were defined as ≤ 1 mm (limb leads) and ≤ 3 mm (chest leads) [26]. CKmb elevation was defined as > 25 IU/L.

### ECG analyses

All ECGs were included in the analyses but those without dates (*n* = 9) were excluded from the time trend analyses. Analyses [Stata v13 (STATA Corporation, College Station, TX] were descriptive. Trends in clinical, laboratory and ECG data are reported by illness day (D) where D1 is the first day of reported fever. For convenience, ECGs performed on D30 or later are grouped as D30. A linear mixed effects model was used to determine the factors associated with changes in the QTcF interval over time (age, sex, illness day, and, over time, heart rate, temperature, serum sodium, potassium and CKmb fraction); only significant (*p* < 0.05) factors identified in the univariate analyses and age were entered into the model.

## Results

### General findings

A total of 136/143 recruited patients had 330 ECGs (235 as inpatients, 95 as outpatients). Their median (range) age was 24 (16–72) years and they were enrolled on median illness D5 (1–8); 62/143 (43%) were females. Several patients had been prescribed vitamins, paracetamol and antibiotics prior to their admission. Six patients reported previous dengue but serology suggested a third each had primary and secondary infections with some 40% remaining indeterminate. Median hospital stay was 5 days, with an interquartile range of 2–12 days [27].

By WHO 2009 criteria, 132 had non-severe severe dengue and 4 severe dengue: shock (systolic blood pressure < 70 mm Hg) and aspartate transaminase (AST) > 1000 IU/L (*n* = 3). For the severe patients, no ECGs were done in one patient, 1 in the shocked patient, and 1 and 3 in the two high AST patients. As these ECGs were unremarkable, they were included in the total ECG pool. At least one WHO defined warning sign was seen in 122/143 (85.3%) patients. For all patients, the median platelet count nadired on illness D6 and the mean peak haematocrit was almost identical on illness D4–6 [24]. No patients required intense cardiac monitoring and there were no deaths.

### ECGs

**Fig. 2:**
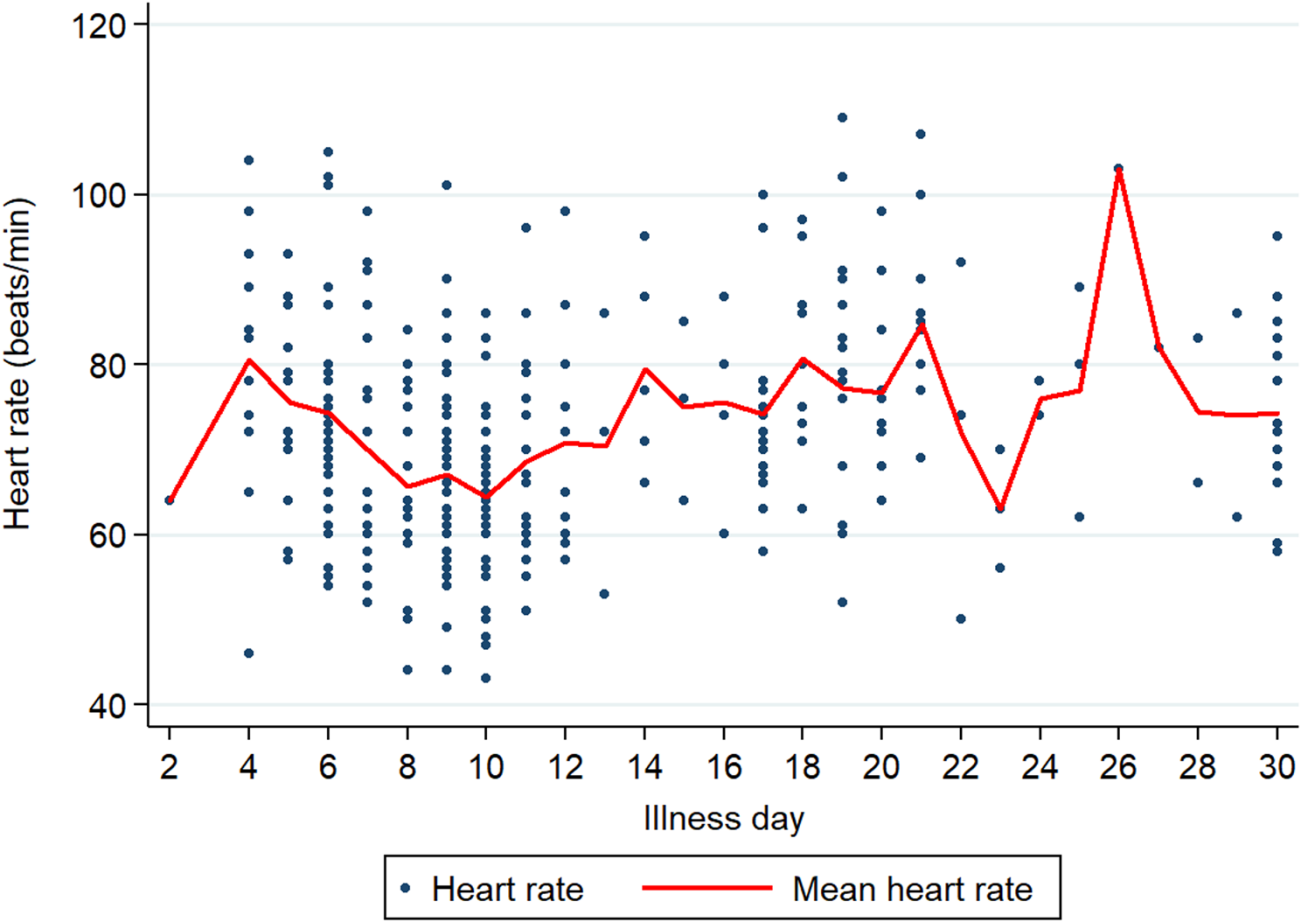
The mean heart rate by illness day

**Fig. 3:**
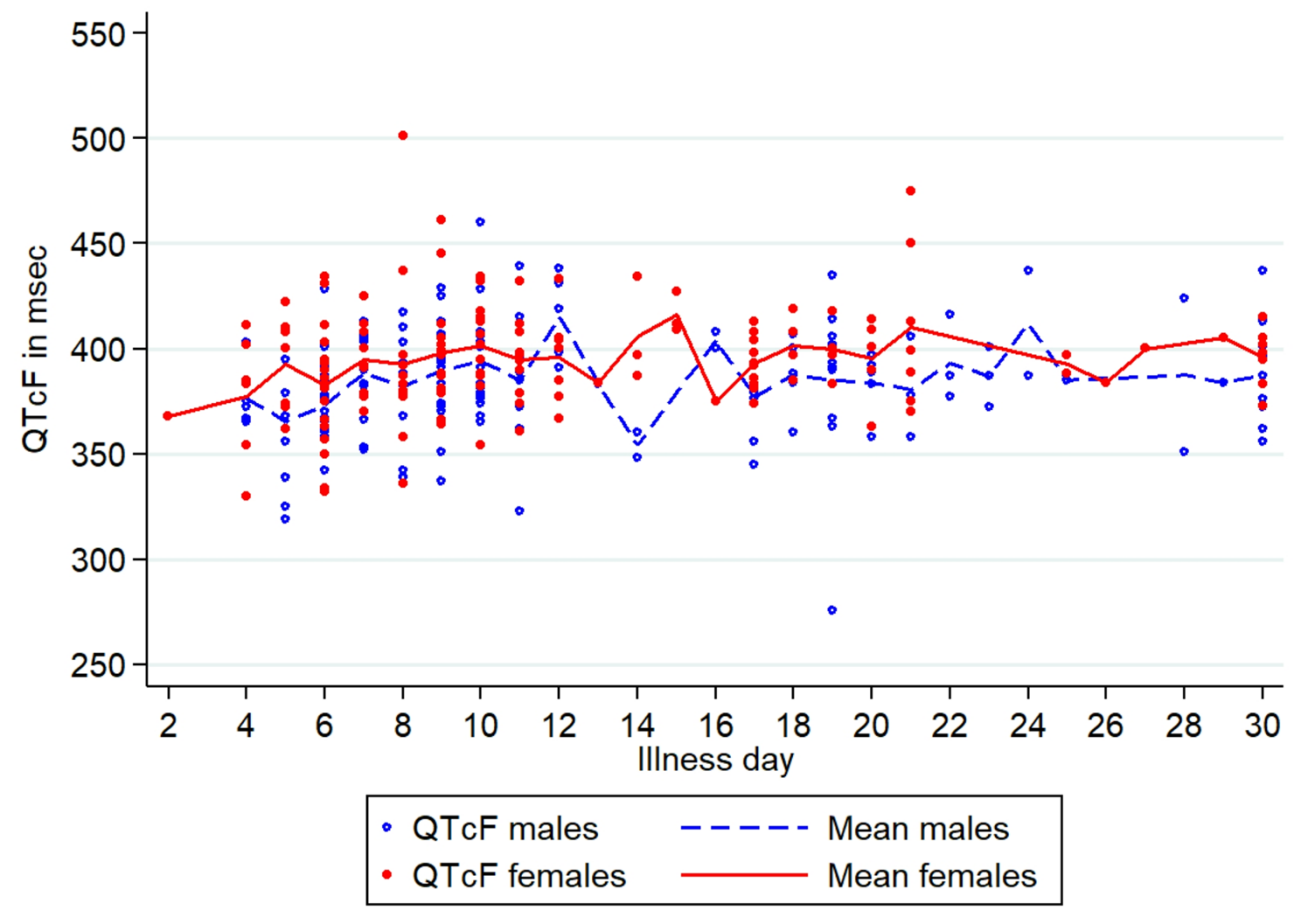
The mean Fridericia corrected QT intervals by illness day in females and males

The mean baseline heart rate, 80/min, declined steadily over time and nadired on D10 for a mean rate of 64.4 (95% CI: 60.9–67.9)/min, the same day as the mean peak QTcF 398.3 ms (95% CI: 390.8–405.8, range 395–460 ms) when almost all patients were afebrile (Figs. 2 and 3, Additional file 1: Fig S1). Across all illness days, the QTcF intervals ranged from 276 to 501 ms for a mean of 390.3 ms (median 391 ms); 3 females (461, 475, 501 ms) and 1 male (460 ms) had prolonged QTcF values.

Only illness day and sex explained the variation in the QTcF over time and both were associated with a greater QTcF interval (Table 1; Fig. 2). Serum potassium values were mostly within the normal range of 3.5–5.0 mmol/L (Additional file 2: Fig S2).

**Table 1:** The relationship between the QTcF and assessed independent factors

Variable	Univariate Analysis		Multivariable Analysis	
	Slope (95%CI)	*p*-value	Slope (95%CI)	*p*-value
Age (years)	-0.029 (-0.352,0.294)	0.862	-0.095(-0.418,0.229)	0.566
Sex: female Male	8.724 (2.182,15.266) Reference	0.009	9.741(2.964,16.517)	0.005
Illness duration (days)	0.500 (0.386,0.614)	< 0.0001	0.502 (0.388,0.616)	< 0.0001
Temperature (^0^C)	0.057 (-0.892,1.005)	0.907	NA	NA
Potassium (mmol/L)	-0.019 (-3.073,3.035)	0.990	NA	NA
Sodium (mmol/L)	-0.002 (-0.232,0.229)	0.989	NA	NA
Creatine kinase MB (IU/L)	0.003 (-0.028,0.033)	0.869	NA	NA

There were 53 episodes of sinus bradycardia (< 60/min) in 39/136 (28.7%) patients and the proportions of bradycardic patients varied by illness day: 1/12 (8.3%) patients ≤D4, peaking at 9/34 (26.5%) on D10, and declining to 3/19 (15.8%) on D30. Sinus tachycardia (> 100/min) was observed 8 times and persisted at follow up in 2 patients.

The PR interval was within normal limits (120–200 ms) for most patients and the mean PR remained steady over time (Additional file 3: Fig S3). Seven episodes of 1⁰ heart block were seen in four patients and was persistent in one. There was no significant correlation between the PR interval and heart rate (aR^2^=0.0004, *p* = 0.287).

In the 330 ECGs, ST segments were either normal, elevated within normal limits (including one patient with type I Brugada syndrome), or depressed in 113 (34.2%), 215 (65.2%) and 2 (0.6%), respectively. ST depression was associated with inverted T waves in leads III, aVF, V1, V3 & V4. T waves were normal in 67 (20.3%) ECGs, peaked in 9 (2.7%), and inverted in 254 (75.4%); the most common lead with inverted T waves was V1 (233/254, 91.2%). Asymptomatic pathological T wave inversion was seen in 12 ECGs in 11 patients from D4–20, which reverted to normal in 7 patients, persisted in 1, and recorded for the first time on the last ECG in 1; ECGs were not done in the other 2 patients. Three patients had evidence of a right bundle branch block (BBB) that was probably idiopathic in 2 because it was present in all 3 ECGs. Two patients had left BBB that was probably dengue related in 1. There was insufficient follow data in 2 patients to determine a cause of their BBBs.

**Fig. 4:**
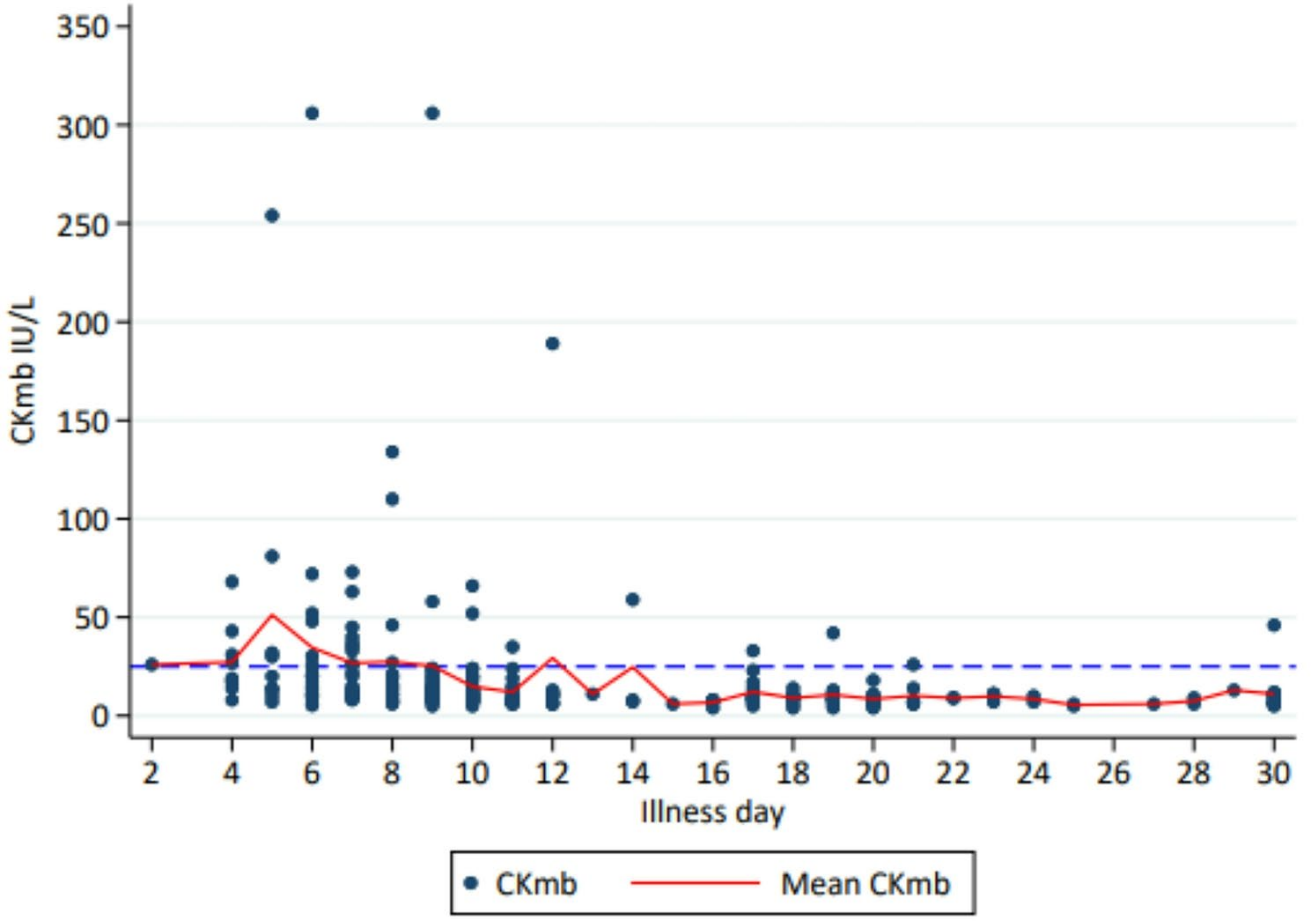
Concentrations of the serum creatine kinase MB fraction over time. 25 IU/L is the upper limit of normal

CKmb concentrations > ULN of 25 IU/L were noted in 64 of 134 patients (47.1%), mostly in the first 10 days of illness (Fig. 4).

## Discussion

In this observational study of predominantly non-severe adult dengue, most ECGs were normal and sinus bradycardia was the most common ECG change. Although there was a correlation between a falling heart rate and increasing QTcF prolongation, heart rate did not significantly explain QTcF variation over time. ECG patterns suggestive of myocardial injury was confined to transient inverted T waves in the inferior and lateral chest leads in < 10% of patients whilst elevated CK-MB concentrations above the upper reference limit were detected in a little under 50%.

Others have also reported mostly normal ECGs and transient nonspecific ECG changes over time in dengue [2, 9, 12, 17, 28]. We observed sinus bradycardia in just over a quarter of patients with the highest proportions of bradycardia occurring on D9 and 10 which persisted up to D30 in a small number, consistent with previous research [6, 8, 9, 19]. The cause of sinus bradycardia in dengue remains unclear but may be related to increased vagal activity on the sinus node [23]. There was a tendency for the mean QTcF to increase over the first 10 days in parallel with a decrease in the mean heart rate but the majority of QTcF values remained within normal limits and, expectedly, females had higher mean values compared to males. In only 5 patients had QTcF prolongation and only 1 female had a QTcF of 501 ms that was transient. These findings are consistent with those of Furlan et al. [17] who reported increases in QTcB in their patients compared to healthy controls. Based on the evidence to date, drug developers should be reassured that non-severe dengue patients appear to be at very low intrinsic risk of Torsades de Pointes consequent to QTc prolongation.

We found that ~ 10% of patients had ECG patterns of cardiac injury, mostly in the form of transient inverted T waves in the inferior and lateral chest leads, occurring on median illness day 9 when patients were recovering; none reported chest pain. We confined the determination of pathological T wave inversion to these leads to avoid the uncertainty of normal variant T wave inversion in other leads. There were no concomitant pathological ST elevation segment changes, another ECG sign of acute myocardial injury, contrasting to the findings of Wali et al. who found concomitant ST elevation and T wave inversion in their series of severe dengue [29]. This difference is likely to be due to differences in dengue severity but also reflects the insensitivity of the ECG to detect myocarditis [30]. Just under half of our patients had a raised CKmb fraction exceeding the ULN and this is broadly consistent with Jadav et al. who reported a little under 40% in their cohort of mostly mild dengue [28]. The CKmb is used as a cardiac biomarker but has less sensitivity and specificity than troponins for diagnosing myocarditis [31]. Indeed, the CKmb is also present at ~ 5 − 7% in skeletal muscle and increases are seen in skeletal muscle injury/disease [32, 33]; therefore, caution is needed in ascribing all cases of raised CKmb to myocarditis. In keeping with published data, we found small numbers of patients with sinus tachycardia, 1st degree AVB and new BBB which were generally transient.

ECG alone is insufficient for detecting myocarditis due to its inherently low sensitivity—estimated at less than 50%—and its non-specific findings [34]; common abnormalities, such as sinus tachycardia or non-specific ST-segment and T-wave changes, are not pathognomonic for myocarditis and often fail to definitively indicate myocardial injury [34]. This diagnostic limitation frequently necessitates further, more specific investigations, including cardiac troponins, or advanced imaging modalities such as echocardiogram and cardiac MRI, to achieve a definitive diagnosis of myocarditis. In the context of dengue fever, several studies report that cardiac manifestations are significantly more prevalent in more severe cases compared to non-severe cases [2, 9, 13, 18]. Moreover, many ECG changes observed in non-severe dengue are transient and benign, and do not correlate with underlying myocarditis or adverse outcomes [11, 17].

In our study, the ECG showed only poor expression of myocardial injury signs among young adults with non-severe dengue. These findings suggest that hospitals should reconsider the necessity of routine ECGs in this population, where their clinical utility for detecting myocarditis appears limited, particularly in resource-limited settings such as Vietnam. This approach is consistent with major international guidelines e.g., WHO 2009 guidelines, which do not explicitly recommend routine ECG monitoring for all patients with non-severe dengue but instead emphasise targeted monitoring for warning signs and progression to severe disease.

Despite the existence of previous studies that have investigated cardiovascular manifestations and ECG changes in dengue disease, our study — as the first to evaluate ECG changes and the role of routine ECG in Vietnam — provides valuable insights that may contribute to the optimisation of cost-effective management strategies for Vietnamese dengue patients, particularly otherwise healthy young adults with non-severe dengue. This is especially relevant in the context of dengue imposing a substantial health and economic burden in Vietnam, with an estimated 2,263,880 symptomatic cases occurring annually, accounting for nearly 40,000 disability-adjusted life years (DALYs) each year and leading to a total annual cost of dengue illness in Vietnam of nearly US$100 million [35, 36].

Our study had several limitations. The number of patients was comparatively low and the small number of ECGs analysed, particularly for the identification of ECG changes over time, was due to the comparatively late presentation of many patients, the absence of some ECG data, and the short follow up period. This limited the statistical power to detect small dynamic changes but we were able to show trends over the short term. The absence of a control group, either healthy individuals or patients with other febrile illnesses, makes it difficult to determine whether the ECG changes observed are specific to dengue infection or represent non-specific responses to febrile illness. This has also been a weakness of many ECG observational studies in dengue. We were unable to assess troponin levels and so evidence consistent with myocarditis rested on the raised CKmb, a less sensitive and specific biomarker compared to troponins.

Patients were otherwise healthy young adults who were not taking concomitant medications; therefore, our results may not be generalisable to older populations with comorbidities or those taking drugs that can prolong the QT interval. In addition, as this is a single-centre study, its findings may not be generalisable to the broader population of Vietnam. The study was not designed to detect possible long-term ECG effects, although most ECG changes were transient.

Finally, as our data were collected several years ago, epidemiological changes, including possible changes in the intrinsic cardiotropism of dengue virus serotypes, may have occurred that might limit the applicability of our findings to the present-day population. Future studies with larger sample sizes, appropriate control groups, and systematic ECG monitoring across all phases of the disease are warranted to address these gaps, supplemented by an economic analysis to determine the benefit of routine ECG screening for non-severe dengue.

## Conclusions

Our small observational study conducted in a busy referral hospital has given us a snapshot of the ECG changes in predominantly young Vietnamese adults with mostly non-severe dengue; these changes were benign, non-specific, and generally transient. Although these findings need to be validated by larger, national-level studies, they suggest that routine ECGs may not contribute significantly to the management of non-severe dengue in otherwise healthy patients and should not be performed unless there are clinical triggers like cardiac symptoms, detection of an abnormal pulse, and clinical deterioration to severe dengue. More ECG research, including cardiac biomarkers and echocardiogram correlations, in dengue will be useful as a monitoring tool in clinical and pathophysiological studies, e.g., to assess further the value of ECGs, and drug safety evaluations of potential anti-dengue drugs, notably to detect QTc prolongation.

## Supplementary Information

Below is the link to the electronic supplementary material.

Supplementary Material 1: Fig S1. The Fridericia corrected QT intervals by illness day in females and males combined; Fig S2. Trend in the mean serum potassium over time; Fig S3. The PR intervals by illness day.

## Data Availability

Deidentified individual participant data will be available after publication to applicants who provide a sound proposal to the Mahidol Oxford Tropical Medicine Research Unit Data Access Committee. They can contact the corresponding author in the first instance.

## Abbreviations

AST: Aspartate transaminase
AVB: Atrioventricular block
BBB: Bundle branch block
CKmb: Creatinine kinase-myocardial band
D: Day
ECG: Electrocardiogram
ECHO: Echocardiogram
IgG: Immunoglobulin G
IgM: Immunoglobulin M
MRI: Magnetic resonance imaging
NHTD: National Hospital for Tropical Diseases
NS1: Non-structural protein 1
PCR: Polymerase chain reaction
ULN: Upper limit of normal
WHO: World Health Organization
