# Supplementary material for: ECG changes and their utility in adult Vietnamese patients with non-severe dengue
Source: BMC Infect Dis. 2026 Feb 26;26:757. doi: 10.1186/s12879-026-12926-2 (PMC13077852; doi:10.1186/s12879-026-12926-2)

**Figure S1. The mean Fridericia corrected QT interval by illness day in females and males combined.**


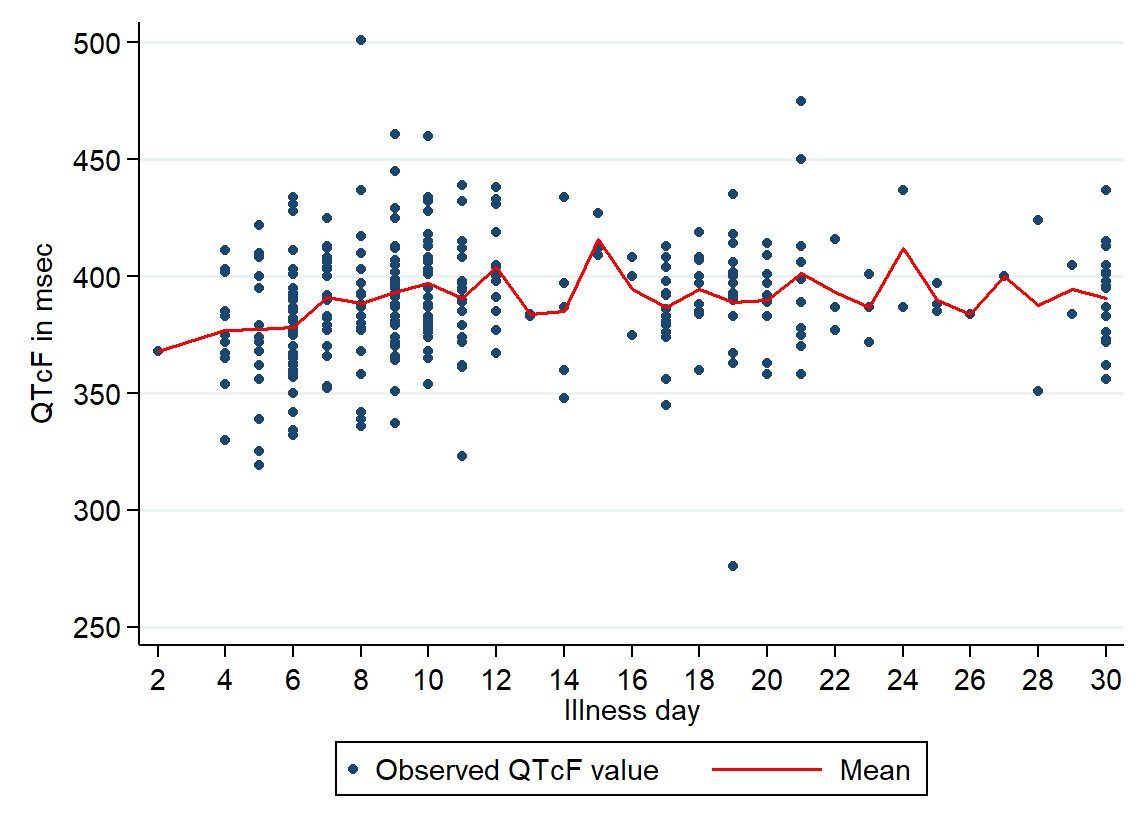


**Figure S2. Trend in the mean serum potassium over time.**


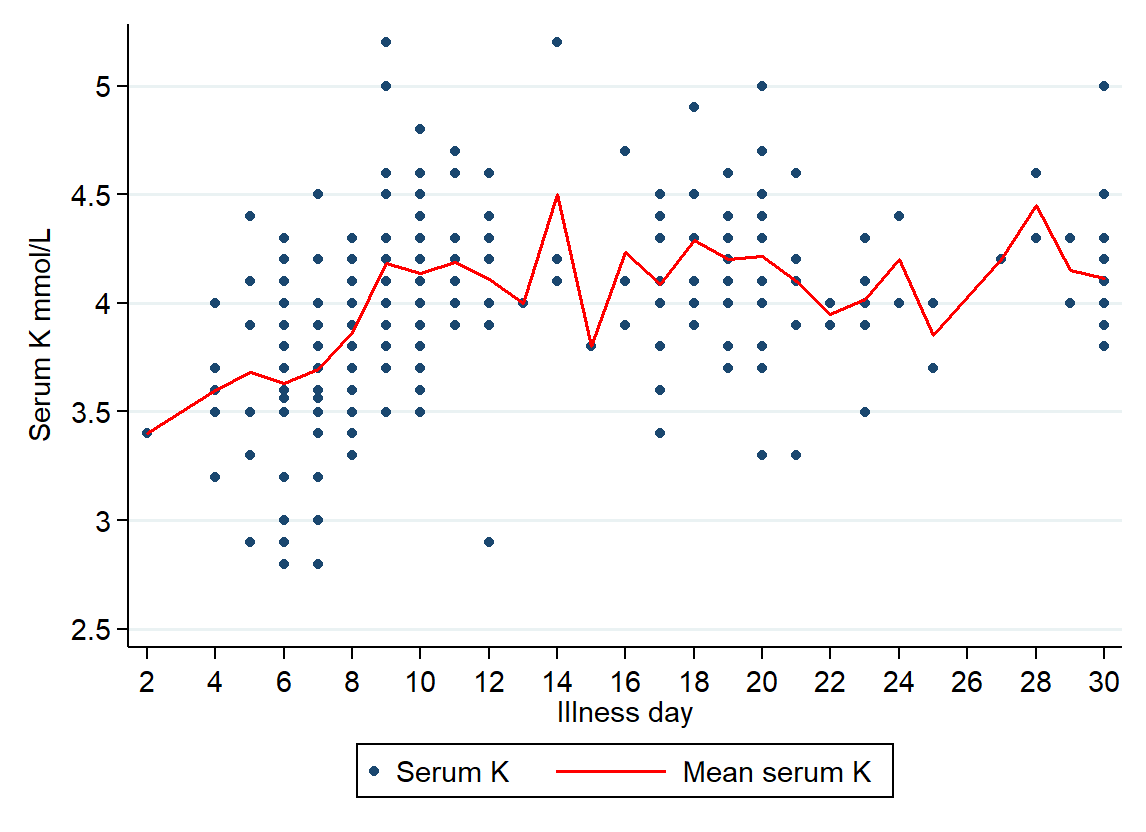


Figure S3. The PR intervals by illness day.


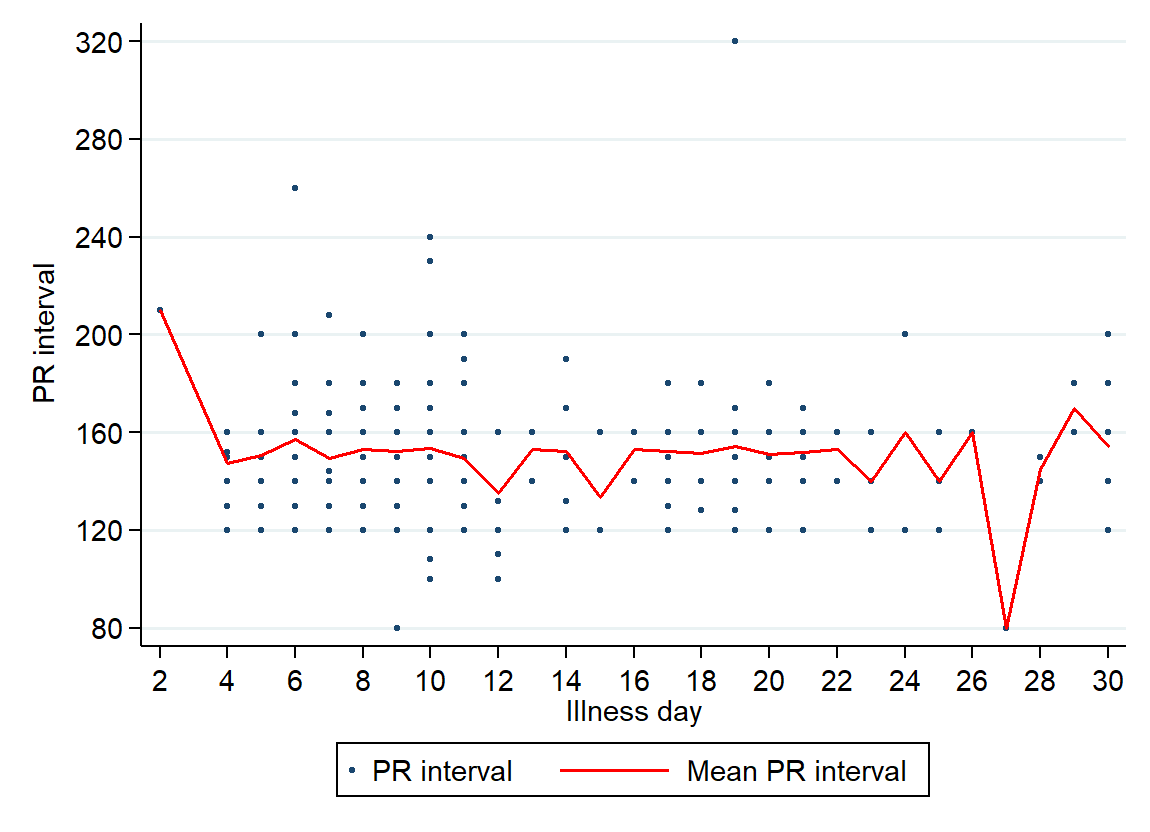

Supplement: Supplementary file 1 — Supplementary Material 1: Fig S1. The Fridericia corrected QT intervals by illness day in females and males combined; Fig S2. Trend in the mean serum potassium over time; Fig S3. The PR intervals by illness day. [file 12879_2026_12926_MOESM1_ESM.docx]
